# Systematic review of applied usability metrics within usability evaluation methods for hospital electronic healthcare record systems: Metrics and Evaluation Methods for eHealth Systems

**DOI:** 10.1111/jep.13582

**Published:** 2021-05-13

**Authors:** Marta Weronika Wronikowska, James Malycha, Lauren J. Morgan, Verity Westgate, Tatjana Petrinic, J Duncan Young, Peter J. Watkinson

**Affiliations:** ^1^ Critical Care Research Group, Nuffield Department of Clinical Neurosciences University of Oxford Oxford UK; ^2^ Department of Acute Care Medicine University of Adelaide Adelaide Australia; ^3^ Nuffield Department of Surgical Sciences University of Oxford, John Radcliffe Hospital Oxford UK; ^4^ Bodleian Health Care Libraries John Radcliffe Hospital, University of Oxford Oxford UK

**Keywords:** electronic health records, electronic patients record (EPR), systematic review, usability methods, usability metrics

## Abstract

**Background and objectives:**

Electronic healthcare records have become central to patient care. Evaluation of new systems include a variety of usability evaluation methods or usability metrics (often referred to interchangeably as usability components or usability attributes). This study reviews the breadth of usability evaluation methods, metrics, and associated measurement techniques that have been reported to assess systems designed for hospital staff to assess inpatient clinical condition.

**Methods:**

Following Preferred Reporting Items for Systematic Reviews and Meta‐Analyses (PRISMA) methodology, we searched Medline, EMBASE, CINAHL, Cochrane Database of Systematic Reviews, and Open Grey from 1986 to 2019. For included studies, we recorded usability evaluation methods or usability metrics as appropriate, and any measurement techniques applied to illustrate these. We classified and described all usability evaluation methods, usability metrics, and measurement techniques. Study quality was evaluated using a modified Downs and Black checklist.

**Results:**

The search identified 1336 studies. After abstract screening, 130 full texts were reviewed. In the 51 included studies 11 distinct usability evaluation methods were identified. Within these usability evaluation methods, seven usability metrics were reported. The most common metrics were ISO9241‐11 and Nielsen's components. An additional “usefulness” metric was reported in almost 40% of included studies. We identified 70 measurement techniques used to evaluate systems. Overall study quality was reflected in a mean modified Downs and Black checklist score of 6.8/10 (range 1–9) 33% studies classified as “high‐quality” (scoring eight or higher), 51% studies “moderate‐quality” (scoring 6–7), and the remaining 16% (scoring below five) were “low‐quality.”

**Conclusion:**

There is little consistency within the field of electronic health record systems evaluation. This review highlights the variability within usability methods, metrics, and reporting. Standardized processes may improve evaluation and comparison electronic health record systems and improve their development and implementation.

## 1 INTRODUCTION

Electronic health record (EHR) systems are real‐time records of patient‐centred clinical and administrative data that provide instant and secure information to authorized users. Well designed and implemented systems should facilitate timely clinical decision‐making.^1^ ^,^ ^2^ However^3^ the prevalence of poorly performing systems suggest the common violation of usability principles.^4^

There are many methods to evaluate system usability.^5^ Usability evaluation methods cited in the literature include user trials, questionnaires, interviews, heuristic evaluation and cognitive walkthrough.^6^, ^7^, ^8^, ^9^ There are no standard criteria to compare results from these different methods^10^ and no single method identifies all (or even most) potential problems.^11^

Previous studies have focused on usability definitions and attributes.^12^, ^13^, ^14^, ^15^, ^16^, ^17^ Systematic reviews in this field often present a list of usability evaluation methods^18^ and usability metrics^19^ with additional information on the barriers and/or facilitators to system implementation.^20^ ^,^ ^21^ However many of these are restricted to a single geographical region,^22^ type of illness, health area, or age group.^23^

The lack of consensus on which methods to use when evaluating usability^24^ may explain the inconsistent approaches demonstrated in the literature. Recommendations exist^25^, ^26^, ^27^ but none contain guidance on the use, interpretation and interrelationship of usability evaluation methods, usability metrics and the varied measurement techniques applied to assess EHR systems used by clinical staff. These are a specific group of end‐users whose system‐based decisions have a direct impact on patient safety and health outcomes.

The objective of this systematic review was to identify and characterize usability metrics (and their measurement techniques) within usability evaluation methods applied to assess medical systems, used exclusively by hospital based clinical staff, for individual patient care. For this study, all components in the included studies have been identified as “metrics” to facilitate comparison of methods when testing and reporting EHR systems development.^28^ In such cases, Nielsen's satisfaction attribute is equivalent to the ISO usability component of satisfaction.

## 2 METHODS

This systematic review was registered with PROSPERO (registration number CRD42016041604).^29^ During the literature search and initial analysis phase, we decided to focus on the methods used to assess graphical user interfaces (GUIs) designed to support medical decision‐making rather than visual design features. We have changed the title of the review to reflect this decision. We followed the Preferred Reporting Items for Systematic Reviews and Meta‐Analyses (PRISMA) guidelines^30^ (Appendix Table S1).

### 2.1 Eligibility criteria

Included studies evaluated electronic systems; medical devices used exclusively by hospital staff (defined as doctors, nurses, allied health professionals, or hospital operational staff) and presented individual patient data for review.

Excluded studies evaluated systems operating in nonmedical environments, systems that presented aggregate data (rather than individual patient data) and those not intended for use by clinical staff. Results from other systematic or narrative reviews were also excluded.

### 2.2 Search criteria

The literature search was carried out by TP using Medline, EMBASE, CINAHL, Cochrane Database of Systematic Reviews, and Open Grey bibliographic databases for studies published between January 1986 and November 2019. The strategy combined the following search terms and their synonyms: usability assessment, EHR, and user interface. Language restrictions were not applied. The reference lists of all included studies were checked for further relevant studies. Appendix Table S2 presents the full Medline search strategy.

### 2.3 Study selection and analysis

The systematic review was organized using Covidence systematic review management software (Veritas Health Innovation Ltd, Melbourne).^31^ Two authors (MW, VW) independently reviewed all search result titles and abstracts. The full text studies were then screened independently (MW, VW). Any discrepancies between the authors regarding the selection of the articles were reviewed by a third party (JM) and a consensus was reached in a joint session.

### 2.4 Data extraction

We planned to extract the following data:

1. Demographics (authors, title, journal, publication date, country).
2. Characteristics of the end‐users.
3. Type of medical data included in EHR systems.
4. Usability evaluation methods and their types, such as:
  - questionnaires or surveys,
  - user trials,
  - interviews,
  - heuristic evaluation.
5. Usability metrics (components variously defined as attributes, criteria,^32^ or metrics^33^). For the purpose of this review, we adopted the term “metric” to describe any such component) but we include all metric‐similar terms used by authors in included studies:
  - satisfaction, efficiency, effectiveness metrics,
  - learnability, memorability, errors components,
6. Types and frequency of usability metric analysed within usability evaluation methods.

We extracted data into two stages. Stage 1 relied on the extraction of general data from each of the studies that met our primary criteria based the original data extraction form. Stage 2 extended the extraction to gain more specific information such as the measurement techniques for each identified metric as we observed that these were reported in different ways.

The extracted data was assessed for agreement reaching the goal of >95%. All uncertainties regarding data extraction were resolved by discussion among the authors.

### 2.5 Quality assessment

We used two checklists to evaluate quality of included studies. First used tool, the Downs & Black (D&B) Checklist for the Assessment of Methodological Quality^34^ contains 27 questions, covering the following domains: reporting quality (10 items), external validity (three items), bias (seven items), confounding (six items) and power (one item). It is widely used for clinical systematic reviews because it is validated to assess randomized controlled trials, observational and cohort studies. However, many of the D&B checklist questions have little or no relevance to studies evaluating EHR systems, particularly because EHR systems are not classified as “interventions.” Due to this fact, we modified D&B checklist to have usability‐oriented tool. The purpose of our modified D&B checklist, constructed of 10 questions, was quality assessment of the aim of the study (specific to usability evaluation methods) evidence that included methods and metrics were supported by peer reviewed literature. Our modified D&B checklist investigated whether the participants of the study were clearly described and representative of the eventual (intended) end‐users, the time period over which the study was undertaken being clearly described and the results reflected the methods and described appropriately. The modified D&B checklist is summarized in the appendix (Appendix Table S3). Using this checklist, we defined “high quality” studies as those which scored well in each of the domains (scores ≥ eight). Those studies, which scored in most but not all domains were defined as “moderate quality” (scores of six and seven). The remainder were defined as “low quality” (scores of five and below). We decided to not exclude any paper due to low quality.

## 3 RESULTS

**FIGURE 1:**
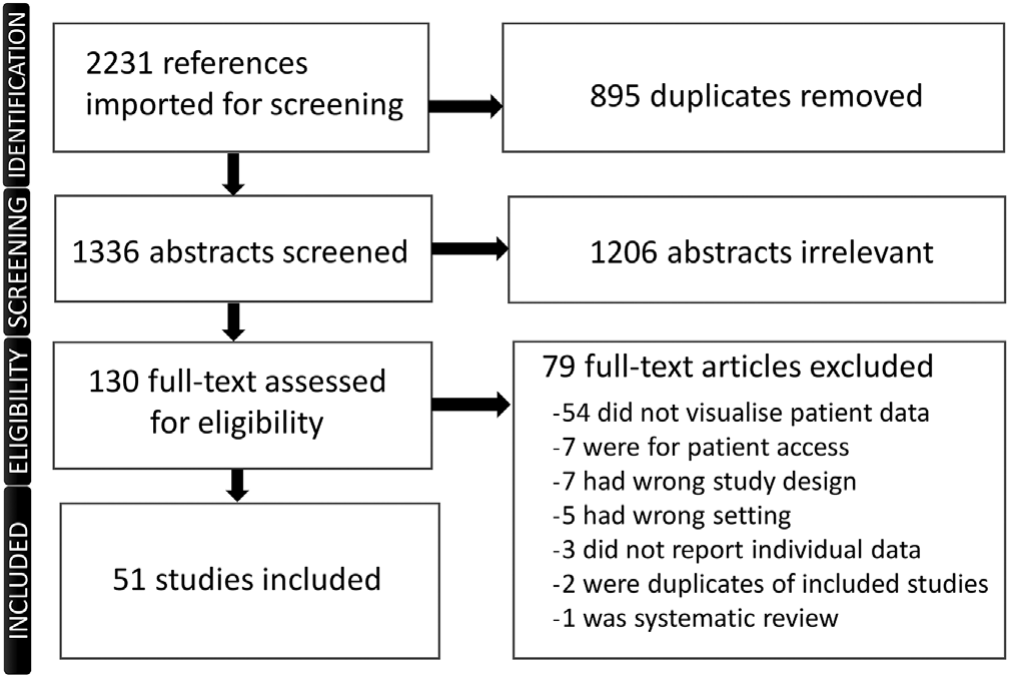
Study selection process: PRISMA flow diagram

We followed the PRISMA guidelines for this systematic review (Appendix Table S1). The search generated 2231 candidate studies. After the removal of duplicates, 1336 abstracts remained (Figure 1). From these, 130 full texts were reviewed, with 51 studies eventually being included. All included studies were published between 2001 and 2019. Of the included studies, 86% were tested on clinical staff, 6% on usability experts and 8% on both clinical staff and usability experts. The characteristics of the included studies are summarized in Table 1.

**TABLE 1:** Details of included studies

Ref	Author	Year	Country	Participants	Number	System type
35	Aakre et al.	2017	USA	Internal Medicine Residents, Resident, Fellows, Attending Physicians	26	EHR with SOFA^a^ score calculator
36	Abdel‐Rahman	2016	USA	Physicians, Residents, Nurses, Pharmacologists, Pharmacists, Administrators	28	EHR with the addition of a medication display
37	Al Ghalayini, Antoun, Moacdich	2018	Lebanon	Family Medicine Residents	13	EHR evaluation
38	Allen et al.	2006	USA	“Experts” experienced in usability testing	4	EHR evaluation
39	Belden et al.	2017	USA	Primary Care Physicians	16	Electronic clinical notes
40	Brown et al.	2001	USA	Nurses	10	Electronic clinical notes
41	Brown et al.	2016	UK	Health Information System Evaluators	8	Electronic quality‐improvement tool
42	Brown et al.	2018	UK	Primary Care Physicians	7	Electronic quality‐improvement tool
43	Chang et al.	2011	USA	Nurses, Home Aides, Physicians, Research Assistants	60	EHR on mobile devices
44	Chang et al.	2017	Taiwan	Medical Students, Physician Assistant Students	132	EHR with the addition of a medication display
45	Devine et al.	2014	USA	Cardiologists, Oncologists	10	EHR with clinical decision support tool
46	Fidler et al.	2015	USA	Critical Care Physicians, Nurses	10	Monitoring – physiology (for patients with arrhythmias)
47	Forsman et al.	2013	Sweden	Specialists Physicians, Resident Physicians, Usability Experts	12	EHR evaluation
48	Fossum et al.	2011	Norway	Registered Nurses	25	EHR with clinical decision support tool
49	Gardner et al.	2017	USA	Staff Physicians, Fellows, Medical Resident, Nurse Practitioners, Physician Assistant	14	Monitoring – physiology (for patients with heart failure)
50	Garvin et al.	2019	USA	Gastroenterology Fellows, Internal Medicine Resident, Interns	20	EHR with clinical decision support tool for patients with cirrhosis
51	Glaser et al.	2013	USA	Undergraduates, Physicians, Registered Nurses	18	EHR with the addition of a medication display
52	Graber et al.	2015	Iran	Physicians	32	EHR with the addition of a medication display
53	Hirsch et al.	2012	Germany	Physicians	29	EHR with clinical decision support tool
54	Hirsch et al.	2015	USA	Internal Medicine Residents, Nephrology Fellows	12	EHR evaluation
55	Hortman, Thompson	2005	USA	Faculty Members, Student Nurse	5	Electronic outcome database display
56	Hultman et al.	2016	USA	Resident Physicians	8	EHR on mobile devices
57	Iadanza et al.	2019	Italy	An evaluator	1	EHR with ophthalmological pupillometry display
58	Jaspers et al.	2008	Netherlands	Clinicians	116	EHR evaluation
59	Kersting, Weltermann	2019	Germany	General Practitioners, Practice Assistants	18	EHR for supporting longitudinal care management of multimorbid seniors
60	Khairat et al.	2019	USA	ICU Physicians (Attending Physicians, Fellows, Residents)	25	EHR evaluation
61	Khajouei et al.	2017	Iran	Nurses	269	Electronic clinical notes
62	King et al.	2015	USA	Intensive Care Physicians	4	EHR evaluation
63	Koopman, Kochendorfen, Moore	2011	USA	Primary Care Physicians	10	EHR with clinical decision support tool for diabetes
64	Laursen et al.	2018	Denmark	Human Computer Interaction Experts, Dialysis Nurses and Nephrologist	8	EHR with clinical decision support tool for patients of need of haemodialysis therapy
65	Lee et al.	2017	South Korea	Professors, Fellows, Residents, Head Nurses, Nurses	383	EHR evaluation
66	Lin et al.	2017	Canada	Physicians, Nurses, Respiratory Therapists	22	EHR evaluation
67	Mazur et al.	2019	USA	Residents and Fellows (Internal Medicine, Family Medicine, Paediatrics Specialty, Surgery, Other)	38	EHR evaluation
68	Nabovati et al.	2014	Iran	Evaluators	3	EHR evaluation
69	Nair et al.	2015	Canada	Family Physicians, Nurse Practitioners, Family Medicine Residents	13	EHR with clinical decision support tool for chronic pain
70	Neri et al.	2012	USA	Genetic Counsellors, Nurses, Physicians	7	Electronic genetic profile display
71	Nouei et al.	2015	Iran	Surgeons, Assistants, Other Surgery Students (Residents Or Fellowship)	unknown	EHR evaluation within theatres
72	Pamplin et al.	2019	USA	Physicians, Nurses, Respiratory Therapists	41	EHR evaluation
73	Rodriguez et al.	2002	USA, Puerto Rico	Internal Medicine Resident Physicians	36	EHR evaluation
74	Schall et al.	2015	France	General Practitioners, Pharmacists, NonClinician E‐Health Informatics Specialists, Engineers.	12	EHR with clinical decision support tool
75	Seroussi et al.	2017	USA	Nurses, Physicians	7	EHR evaluation
76	Silveira et al.	2019	Brasil	Cardiologists and Primary Care Physicians	15	EHR with clinical decision support tool for patients with hypertension
77	Su et al.	2012	Taiwan	Student Nurses	12	EHR evaluation
78	Tappan et al.	2009	Canada	Anaesthesiologists, Anaesthesia Residents	22	EHR evaluation within theatres
79	Van Engen‐Verheul et al	2016	Netherlands	Nurses, Social Worker, Medical Secretary, Physiotherapist	9	EHR evaluation
80	Wachter et al.	2003	USA	Anaesthesiologists, Nurse Anaesthetists, Residents, Medical Students	46	Electronic pulmonary investigation results display
81	Wu et al.	2009	Canada	Family Physicians, Internal Medicine Physician	9	EHR on mobile devices
82	Zhang et al.	2009	USA	Physicians, Health Informatics Professionals	8	EHR evaluation
83	Zhang et al.	2013	USA	Physicians, Health Informatics Professionals	unknown	EHR evaluation
84	Zheng et al.	2007	USA	Active Resident Users, Internal Medicine Residents	30	EHR with clinical reminders
85	Zheng et al.	2009	USA	Residents	30	EHR with clinical reminders

^a^ Sequential Organ Failure Assessment.

Of the included studies, 16 evaluated generic EHR systems. Eleven evaluated EHR decision support tools (four for all ward patients, one for patients with diabetes, one for patients with chronic pain, one for patients with cirrhosis, one for patients requiring haemodialysis therapy, one for patients with hypertension, one for cardiac rehabilitation and one for management of hypertension, type‐2 diabetes and dyslipidaemia). Seven evaluated specific electronic displays (physiological data for patients with heart failure, arrhythmias, also genetic profiles, an electronic outcomes database, longitudinal care management of multimorbid seniors, chromatic pupillometry data, and pulmonary investigation results).

Four studies evaluated medication specific interfaces. Three evaluated electronic displays for patients' clinical notes. Three studies each evaluated mobile EHR systems. Two evaluated EHR systems with clinical reminders. Two evaluated quality improvement tools. Two evaluated systems for use in the operating theatre environment and one study evaluated a sequential organ failure assessment score calculator to quantify the risk of sepsis.

We extracted data on GUIs. All articles provided some description of GUIs, but these were often incomplete, or were a single screenshot. It was not possible to extract further useful information on GUIs. Appendix Table S4 presents the specification of type of data included in EHR systems.

### 3.1 Usability evaluation methods

Ten types of methods to evaluate usability were used in the 51 studies that were included in this review. These are summarized in Table 2. We categorized the 10 methods into broader groups: user trials analysis, heuristic evaluations, interviews and questionnaires. Most authors applied more than one method to evaluate electronic systems. User trials were the most common method reported, used in 44 studies (86%). Questionnaires were used in 40 studies (78%). Heuristic evaluation was used in seven studies (14%) and interviews were used in 10 studies (20%). We categorized thinking aloud, observation, a three‐step testing protocol, comparative usability testing, functional analysis and sequential pattern analysis as user trials analysis. Types of usability evaluation methods are described in Table 3.

**TABLE 2:** Usability evaluation methods

		User trial analysis									
Ref	User trial	Thinking aloud	Observation	Comparative usability testing	A three step testing protocol	Functional analysis	Sequential pattern analysis	Cognitive walkthrough	Heuristic evaluation	Questionnaire / Surveys	Interview
35	*		*							*	
36	*	*	*					*		*	
37	*	*	*							*	
38									*		
39	*			*						*	
40	*									*	
41								*	*		
42	*		*							*	*
43	*									*	
44	*									*	
45	*	*							*	*	
46	*		*							*	
47	*	*	*							*	*
48	*	*	*					*		*	*
49	*	*								*	
50	*									*	*
51	*									*	
52	*									*	
53	*									*	
54	*	*								*	
55	*	*	*							*	
56	*	*								*	
57									*		
58	*									*	
59	*	*								*	*
60	*									*	*
61	*									*	
62	*	*								*	*
63	*	*	*								*
64	*	*							*		
65	*									*	
66	*	*	*							*	
67	*									*	
68									*		
69	*	*	*							*	
70	*	*	*							*	
71	*									*	*
72	*	*								*	*
73	*									*	
74	*	*								*	
75	*	*								*	
76	*									*	
77	*	*								*	
78	*	*								*	
79	*	*							*		
80	*				*					*	
81	*	*	*								
82						*					
83	*									*	
84							*				
85							*				
N	**44**	**23**	**13**	**1**	**1**	**1**	**2**	**3**	**7**	**40**	**10**
%	**86**	**45**	**25**	**2**	**2**	**2**	**4**	**6**	**14**	**78**	**20**

**TABLE 3:** Description of the methods included as User Trials Analysis

Method	Description	References
User trial	A process through which end‐users (or potential end‐users) complete tasks using the system under evaluation. Every participant should be aware of the purpose of the system and analysis. According to Neville et al.^35^ participants should be “walked through” through the task under analysis One of the main objectives for a user trial is to collect observation data but sometimes information comes from the post‐test interviews or questionnaires.	35 Studies using user trials are indicated in Table 2
Thinking aloud	Verbal reporting method that generates information on the cognitive processes of the user during task performance. The user must verbalize their thoughts as they interact with the interface	36,37,38,39,40,41,42,43,44,45,46,47, 48, 49, 50, 51
Observation	Direct and remote observation of users interacting with the system	52
Comparative Usability Testing	Examines the time to acquire information and accuracy of information	53
Three Step Testing Protocol	Tests for intuitiveness within the system. Step one asks users to identify relevant features within the interface. Step two requires users to connect the clinical variables of interest. Step three asks users to diagnose clinical events based on the emergent features of the display	54
Functional Analysis	Measures “functions” within the EHR and classifies them into either Operations or Objects. Operations are then sub classified into Domains or Overheads.	55, 56
Sequential Pattern Analysis	Searches for recurring patterns within a large number of event sequences. Designed to show “combinations of events” appearing consistently, in chronological order and then in a recurring fashion.	57, 58
Cognitive Walkthrough	Walkthrough of a scenario with execution of actions that could take place during completion of the task completion with expression of comments about use of the interface. It measures ease of learning for new users.	8, 36, 39, 59, 60, 61, 62
Heuristic evaluation	Method that helps to identify usability problems using a checklist related to heuristics. Types of HE methods are reported in Appendix Table S5.	7,37, 49, 59, 63, 64, 65, 66,67, 68
Questionnaire/ Survey	Research instrument used for collecting data from selected group of respondents. The questionnaires used in studies included in this review are summarized in Appendix Table S7.	Appendix Table S7
Interview	Structured research method, which may be applied before the user‐trial, in the middle of user trials or after the user trial. We identified six types of interviews (follow‐up, unstructured, prestructured, semi‐structured, contextual and post‐test interviews), described in Appendix Table S6. The purpose of interviews (unstructured, follow‐up and semi‐structured), applied before the user trial, was understanding the end‐users' needs, their environment, information/communication flow and identification of possible changes, which could improve the process/workflow. The goal of interviews (contextual), applied during user trial, was end‐users observation while they work to collect information about potential utility of systems. The purpose of interviews (prestructured, posttest, semi‐structured), applied after the user trial, was mainly gathering information about missing data, system's weaknesses, opportunities for improvements and users' expectations toward further system's development.	38, 39, 42, 43, 69, 70, 71, 72, 73, 74

Three heuristic evaluation methods were used in seven of the included studies. Four studies used the method described by Zhang et al.^75^ One study, despite application of this method, also used the seven clinical knowledge heuristics outlined by Devine et al.^37^ The three remaining studies used the heuristic checklist introduced by Nielsen.^67^, ^68^ The severity rate scale was sometimes used to judge the importance or severity of usability problems.^76^ Findings from heuristics analyses are summarized in Appendix Table S5.

Six types of interviews were used in 10 (20%) studies. The interviews were carried out before the user trial, in the middle of user trial or after the user trial.

The purpose of interviews (unstructured,^38^ follow‐up,^38^ and semi‐structured^38^) before the user trial was to understand the end‐users' needs, their environment, information/communication flow, and identification of possible changes.

The purpose of interviews (contextual^73^) during the user trial was observation by the end‐users while using the system to collect information about potential system utility.

The purpose of interviews following the user trial (prestructured,^71^ posttest,^38^ semi‐structured^39^ ^,^ ^70^ ^,^ ^72^ ^,^ ^42^, ^43^ ^,^ ^74^ [one called in‐depth debriefing semi‐structured interview^69^]) was mainly gathering information about missing data, system's weaknesses, opportunities for improvements, and users' expectations toward further system development.

Findings from interviews are summarized in Appendix Table S6.

Among the questionnaires, the System Usability Scale (SUS) was used in 16 studies, the Post‐Study System Usability Questionnaire (PSSUQ) was used in five studies, the Questionnaire of User Interaction Satisfaction (QUIS) was used in four studies, the Computer Usability Satisfaction Questionnaire (CSUQ) was used three times and the NASA‐Task Load (NASA‐TLX) was used in six studies. The questionnaires used in studies included in this review are summarized in Appendix Table S7.

### 3.2 Usability metrics

The usability metrics are summarized in Table 4. Satisfaction was measured in 38 studies (75%), efficiency was measured in 32 studies (63%), effectiveness was measured in 31 studies (61%), learnability was measured in 12 studies (24%), errors was measured in 16 studies (31%), memorability was measured in one study (2%) and usefulness metric that was measured in 20 studies (39%).

**TABLE 4:** Usability metrics

Ref	Satisfaction	Efficiency	Effective‐ness	Learn‐ability	Memor‐ability	Errors	Useful‐ness	Total
77		*						1
36	*	*	*	*			*	5
78	*	*	*			*		4
63					*			1
53	*	*	*					3
79	*	*	*					3
59	*		*			*		3
69	*	*	*			*	*	5
80	*	*	*				*	4
81	*	*		*			*	4
37	*	*	*			*	*	5
82	*	*		*		*		4
38	*	*	*	*				4
39	*	*	*				*	4
83	*			*			*	3
70	*	*	*	*			*	5
84	*	*	*					3
85	*	*	*			*	*	5
86		*	*					2
40	*	*	*					3
87	*		*	*		*		4
41	*	*	*					3
64						*		1
60	*	*		*				3
71								0
72	*	*					*	3
88	*							1
42	*							1
43	*	*	*			*		4
65								0
89	*		*				*	3
44	*	*	*			*	*	5
90		*	*			*		3
66			*			*		2
45	*			*				2
46	*	*	*			*	*	5
73	*						*	2
74	*	*	*					3
91	*	*	*	*				4
48	*	*	*	*		*	*	6
92	*						*	2
93						*	*	2
47	*	*	*	*				4
94	*	*	*				*	3
49		*	*			*		3
54	*							1
50	*	*	*				*	4
55	*	*	*				*	4
95								0
57								0
58								0
Total	**38**	**32**	**31**	**12**	**1**	**16**	**20**	
%	**75**	**63**	**61**	**24**	**2**	**31**	**39**	

### 3.3 usability metrics within usability evaluation methods

Table 5 summarizes the variety of usability evaluation methods used to quantify the different metrics. Some authors used more than one method within the same study (e.g., user trial and a questionnaire) to assess the same metric.

**TABLE 5:** Usability metrics and the usability methods used to measure them. Values are the number of studies

	User Trials	Heuristic Evaluation	Interviews	Questionnaires
Satisfaction	10	1	2	31
Efficiency	29	0	0	2
Effectiveness	29	0	0	2
Learnability	4	0	0	10
Memorability	0	1	0	0
Errors	11	5	1	1
Usefulness	5	0	4	11

Satisfaction and errors: These were assessed using all four categories of usability evaluation methods. Satisfaction (analysed in 38 studies) was measured using questionnaires (in 31 studies), user trials (in 10 studies), interviews (in two studies) and heuristic evaluation (in one study).

The most frequently reported metrics of user trials were efficiency and effectiveness (both used in 29 studies). For heuristic evaluation it was errors, for interviews' it was usefulness (in four studies)and for questionnaires it was satisfaction (in 31 studies) and usefulness (in 11 studies).

Results were reported in different ways regardless of types of usability evaluation methods or types of usability metric applied, so we created a list of measurement techniques.

### 3.4 Usability metrics' measurement techniques

We found that different measurement techniques (MT) were used to report the metrics. The number of measurement techniques used to report the identified usability metrics differed from 1 to 25 per single metric. Appendix Table S8 presents all types of measurement techniques applied for all identified metrics and how the measurement technique was used (e.g., within a user trial, survey/questionnaire, interview or heuristic evaluation). The greatest variety in usability metric reporting was found in the case of Nielsen's errors quality component (23 measurement techniques were used), ISO 9001 effectiveness (15 measurements techniques used) and our newly identified usefulness metric (12 measurement techniques used).

User errors, reported using 23 different measurement techniques, were most often reported as the number of errors (*n* = 4) or percentage of errors made (*n* = 6). Authors sometimes provided contextual information about the type of errors (*n* = 5), or reason for errors (*n* = 1). These measurement techniques were investigated within user trials.

The effectiveness metric was reported with 15 measurement techniques. The most frequent ones used were: number of successfully completed tasks (in eight studies), percentage of correct responses (in four studies) and the percentage of participants able to complete tasks (in three studies).

Efficiency was mostly reported as time to complete tasks (*n* = 27). Sometimes this was reported as a comparator against an alternative system (*n* = 13). Task completion was also measured by number of clicks (*n* = 11). Five studies measured the number of clicks compared to a predetermined optimal path. In two cases the time of assessing the patient's state was also measured.

Satisfaction was reported by eight measurement techniques. This was most frequently by questionnaire results (*n* = 31), by general user comments related to the system satisfaction (*n* = 10), by recording the number of positive comments (*n* = 4) or the number of negative comments (*n* = 4) or users preferences across two tested system versions (*n* = 1).

The usefulness metric was reported using 12 different measurement techniques. These included users' comments regarding the utility of the system in clinical practice (*n* = 5), comments about usefulness of layout (*n* = 1), average score of system usefulness (*n* = 5), and total mean scores for work system‐useful‐related dimensions (*n* = 1).

### 3.5 Quality assessment

Results for the quality assessment are summarized in the appendix (Appendix Table S9). We did not exclude articles due to poor quality. For the D&B quality assessment, the mean score (out of a possible 32 points) was 9.9 and the median and mode score were 10. The included studies scored best in the reporting domain, with seven out of the 10 questions generating points. Studies scored inconsistently (and generally poorly) in the bias and confounding domains and no study scored points in the power domain (Appendix Table S10).

Using the Modified D&B checklist the mean score was 6.8 and the median was 7.0 out of a possible 10 points. Seventeen studies (33%) were classified as “high‐quality” (scoring eight or higher), 26 studies (51%) were “moderate‐quality” (scoring six or seven), and the remaining eight studies (16%) were “low‐quality” (scoring five or below). The relationship between the two versions of the D&B scores is shown in the appendix (Appendix Figure S1).

## 4 DISCUSSION

### 4.1 Main findings

This review demonstrates wide variability in both methodological approaches and study quality in the considerable amount of research undertaken to evaluate EHR systems. EHR systems, despite being expensive and complex to implement, are becoming increasingly important in patient care.^96^ Given the pragmatic, rather than experimental nature of EHR systems, it is not surprising that EHR systems evaluation requires an observational or case‐controlled study. Common methodological failings were unreferenced and incorrectly named usability evaluation methods, discrepancies between study aims, methods and results (e.g., authors did not indicate their intention to measure certain metrics and then subsequently reported these metrics in the results or described the usability evaluation methods in method section but did not present the results).

In the future, well‐conducted EHR system evaluation requires established human‐factor engineering driven evaluation methods. These need to include clear descriptions of study aims, methods, users and time‐frames. The Medicines and Healthcare Regulation Authority (MHRA) requires this process for medical devices and it is logical that a comparable level of uniform evaluation may benefit EHRs.^97^

### 4.2 Strengths

We have summarized the usability evaluation methods, metrics, and measurement techniques used in studies evaluating EHR systems. To our knowledge this has not been done before. Our results' tables may therefore be used as a goal‐oriented matrix, which may guide those requiring a usability evaluation method, usability metric, or combination of each, when attempting to study a newly implemented electronic system in the healthcare environment. We identified usefulness as a novel metric, which we believe has the potential to enhance healthcare system testing. Our modified D&B quality assessment checklist was not validated but has the potential to be developed into a tool better suited to assessing studies that evaluate medical systems. By highlighting the methodological inconsistencies presented by researchers in this field we hope to improve the quality of research in the field, which may in turn lead to better systems being implemented in clinical practice.

### 4.3 Limitations

The limitations of the included studies were reflected in the quality assessment: none of the included studies scored >41% in the original D&B checklist, which is indicative of poor overall methodological quality. Results from the modified D&B quality assessment scale, offered by our team, were better but still showed over half the studies were of low or medium quality. A significant proportion of the current research into EHR systems usability has been conducted by commercial, nonacademic entities. These groups have little financial incentive to publish their work unless the results are favourable, so although this review may reflect publication bias, it is unlikely to reflect all current practices. It was sometimes difficult to extract data on the methods used in studies included in this review. This may reflect a lack of consensus on how to conduct studies of this nature, or a systematic lack of rigour in this field of research.

## 5 CONCLUSION

To our knowledge, this systematic review is the first to consolidate applied usability metrics (with their specifications) within usability evaluation methods to assess the usability of electronic health systems used exclusively by clinical staff. This review highlights the lack of consensus on methods to evaluate EHR systems' usability. It is possible that healthcare work efficiencies are hindered by the resultant inconsistencies.

The use of multiple metrics and the variation in the ways they are measured, may lead to flawed evaluation of systems. This in turn may lead to the development and implementation of less safe and effective digital platforms.

We suggest that the main usability metrics as defined by ISO 9241‐1 (efficiency, effectiveness, and satisfaction) used in combination with usefulness, may form part of an optimized method for the evaluation of electronic health systems used by clinical staff. Assessing satisfaction via reporting the users positive and negative comments; assessing efficiency via time to task completion and time taken to assess the patient state; assessing effectiveness via number/percentage of completed tasks and quantifying user errors; and assessing usefulness via user trial with think‐aloud methods, may also form part of an optimized approach to usability evaluation.

Our review supports the concept that high performing electronic health systems for clinical use should allow successful (effective) and quick (efficient) task completion with high satisfaction levels and they should be evaluated against these expectations using established and consistent methods. Usefulness may also form part of this methodology in the future.

## CONFLICT OF INTEREST

The authors declare that they have no competing interests.

## ETHICS STATEMENT

Ethical approval is not required for the study.

## AUTHORS' CONTRIBUTIONS

MW, LM and PW designed the study, undertook the methodological planning and led the writing. TP advised on search strategy and enabled exporting of results. JM, VW and DY assisted in study design, contributed to data interpretation, and commented on successive drafts of the manuscript. All authors read and approved the final manuscript.

## Supporting information


**Appendix Figure S1** Comparative performance of Downs & Black and Modified Downs & Black Quality Assessment checklists
x‐axis: reference number
y‐axis: score (%) of each checklist

**Appendix Table S1** Preferred Reporting Items for Systematic Review and Meta‐Analysis (PRISMA)
*From*: Moher D, Liberati A, Tetzlaff J, Altman DG, The PRISMA Group (2009). Preferred Reporting Items for Systematic Reviews and Meta‐Analyses: The PRISMA Statement. PLoS Med 6 (7): e1000097. doi:10.1371/journal.pmed1000097
For more information, visit: www.prisma-statement.org.

**Appendix Table S2** Search Strategy

**Appendix Table S3** Modified Downs & Black Quality Assessment Checklist

**Appendix Table S4** Information on GUI: type of data included in electronic health record systems

**Appendix Table S5** Heuristic evaluation

**Appendix Table S6** Interviews

**Appendix Table S7 SUS** = System Usability Scale, **PSSUQ** = Post‐Study System Usability Questionnaire, **QUIS** = User Interaction Satisfaction Questionnaire, **CSUQ** = Computer Usability Satisfaction Questionnaire, **SEQ** = a Single Ease Question, **OAIQ** = Object‐Action Interface Questionnaire, **QQ** = Qualitative Questionnaire, **USQ** = User Satisfaction Questionnaire, **SUSQ** = Subjective User Satisfaction Questionnaire, **TAM** = TAM, **PTSQ** = Post‐Task Satisfaction Questionnaire, **PTQ** = Post‐Test Questionnaire, **UQ** = Usability Questionnaire, **UEQ** = Usability Evaluation Questionnaire, **PQ** = Physician's Questionnaire, **TPBT** = Three paper‐based tests, **10 item SQ** = 10‐item Satisfaction Questionnaire, **NASA** = NASA Task Load Index, **PUS** ‐ Perceived Usability Scale, **CQ** = Clinical Questionnaire, **Lee et al Quest** = Questionnaire without name in Lee et al. 2017, **2sets of quest** = Two sets of questionnaires in Zheng et al. 2013, **InterRAI MDS‐HC 2.0** = InterRAI MDS‐HC 2.0, **EHRUS** = the Electronic Health Record Usability Scale, **SAQ** = self‐administered questionnaire, **PVAS** = post‐validation assessment survey, **5pS** ‐usability score ‐ 5‐point scale, **CSS** = The Crew Status Survey

**Appendix Table S8** How usability metrics results were reported ‐ with given number of studies, which used the selected measurement techniques
**S/Q** ‐ Survey/Questionnaire, **UT** ‐ User Trial, **CW‐HE** ‐ Cognitive Walkthrough, **I** ‐ Interview,

**Appendix Table S9** Quality Assessment results (in %) using the Downs & Black checklists

**Appendix Table S10** Domains within the Downs & Black Checklist
The % score of each included study for each domain.

## Data Availability

The data that supports the findings of this study are available in the supplementary material of this article
