# Supplementary material for: Systematic review of applied usability metrics within usability evaluation methods for hospital electronic healthcare record systems: Metrics and Evaluation Methods for eHealth Systems
Source: J Eval Clin Pract. 2021 May 13;27(6):1403–16. doi: 10.1111/jep.13582 (PMC9438452; doi:10.1111/jep.13582)
Supplement: Supplementary file 1 — Appendix Figure S1 Comparative performance of Downs & Black and Modified Downs & Black Quality Assessment checklists x‐axis: reference number y‐axis: score (%) of each checklist [file JEP-27-1403-s007.docx]

**Appendix Figure 1.** Comparative performance of Downs & Black and Modified Downs & Black Quality Assessment checklists

x-axis: reference number

y-axis: score (%) of each checklist
