# Supplementary material for: Systematic review of applied usability metrics within usability evaluation methods for hospital electronic healthcare record systems: Metrics and Evaluation Methods for eHealth Systems
Source: J Eval Clin Pract. 2021 May 13;27(6):1403–16. doi: 10.1111/jep.13582 (PMC9438452; doi:10.1111/jep.13582)
Supplement: Supplementary file 3 — Appendix Table S2 Search Strategy [file JEP-27-1403-s004.docx]

**Appendix Table 2.** Search Strategy

| Search strategy for MEDLINE database |
| --- |
| 1. USER-COMPUTER INTERFACE/ |
| 1. “user interface*”.ti,ab |
| 1. “graphical user interface*”.ti,ab |
| 1. “dashboard*”.ti,ab |
| 1. 1 OR 2 OR 3 OR 4 |
| 1. COMPUTER GRAPHICS/ |
| 1. “computer graphic*”.ti,ab |
| 1. “information visualization*”.ti,ab |
| 1. “data visualization*”.ti,ab |
| 1. “visual analytic*”.ti,ab 2. “data interpretation*” .ti.ab |
| 1. 6 OR 7 OR 8 OR 9 OR 10 OR 11 |
| 1. MEDICAL RECORD SYSTEMS, COMPUTERIZED/ |
| 1. “medical record*”.ti,ab |
| 1. “patient record*”.ti,ab |
| 1. “Integrated information”.ti,ab |
| 1. ELECTRONIC HEALTH RECORDS/ |
| 1. “electronic health record*”.ti,ab 2. “personal health record*”.ti.ab. |
| 1. “electronic medical record*”.ti,ab |
| 1. “charting system*”.ti,ab 2. “risk calculator*” .ti.ab 3. “early warning score*” .ti.ab 4. “clinical referral*” .ti.ab 5. “referral process* ”.ti.ab |
| 1. 13 OR 14 OR 15 OR 16 OR 17 OR 18 OR 19 OR 20 OR 21 OR 22 OR 23 OR 24 OR 25 |
| 1. MEDICAL INFORMATICS/ |
| 1. “big data”.ti,ab |
| 1. “healthcare data”.ti,ab |
| 1. “health care data”.ti,ab |
| 1. (“medical informatics” AND application*).ti,ab |
| 1. 27 OR 28 OR 29 OR 30 OR 31 |
| 1. usability.ti,ab |
| 1. “usability evaluation”.ti,ab |
| 1. Evaluation.ti,ab |
| 1. 33 OR 34 OR 35 |
| 1. 5 OR 12 |
| 1. 26 OR 32 |
| 1. 37 AND 38 |
| 1. 36 AND 39 |
| 1. 40 [limited to:Publication Year 1986-2019] |
