# Supplementary material for: Systematic review of applied usability metrics within usability evaluation methods for hospital electronic healthcare record systems: Metrics and Evaluation Methods for eHealth Systems
Source: J Eval Clin Pract. 2021 May 13;27(6):1403–16. doi: 10.1111/jep.13582 (PMC9438452; doi:10.1111/jep.13582)
Supplement: Supplementary file 4 — Appendix Table S3 Modified Downs & Black Quality Assessment Checklist [file JEP-27-1403-s009.docx]

**Appendix Table 3.** Modified Downs & Black Quality Assessment Checklist

|  | **Questions** | **Give 1 point if:** |
| --- | --- | --- |
| 1 | Is the hypothesis and aim/objective of the study clearly described? | Hypothesis and aim/objectives all clearly described |
| 2 | Are the main usability evaluation methods described in the Introduction or Methods section? | Usability evaluation methods clearly described in Introduction or Methods section |
| 3 | Are all the usability evaluation methods referenced? | Referenced the method if the method is not novel or described the method clearly if the method is novel. |
| 4 | Are the characteristics of the participants participating in the usability evaluation method/s clearly described? | The number of participants, individual job descriptions |
| 5 | Are the participants participating in the usability evaluation method/s representative of the intended eventual users? | The participants and the intended users need to be described |
| 6 | Is the time period described over which the study was carried out? | Time period described |
| 7 | Do the results accurately reflect the methods described? | The authors do exactly what they said they would do. |
| 8 | Are all the studied usability evaluation method results described (numerically or graphically)? | Usability evaluation method results clearly described |
| 9 | Are the usability evaluation methods measured against referenced or defined metrics in sequential testing? | A metric was used to describe user interface performance |
| 10 | Do the authors characterise the results (specific to usability evaluation methods) with the appropriate qualitative/quantitative statistics? | Application of statistics in case of qualitative OR all studies that applied quantitate methods |
