# Supplementary material for: Systematic review of applied usability metrics within usability evaluation methods for hospital electronic healthcare record systems: Metrics and Evaluation Methods for eHealth Systems
Source: J Eval Clin Pract. 2021 May 13;27(6):1403–16. doi: 10.1111/jep.13582 (PMC9438452; doi:10.1111/jep.13582)
Supplement: Supplementary file 5 — Appendix Table S4 Information on GUI: type of data included in electronic health record systems [file JEP-27-1403-s003.docx]

**Appendix Table 4.** Information on GUI: type of data included in electronic health record systems

| **Data type** | **No. of studies** |
| --- | --- |
| Abdominal pain | 2 |
| Activities of daily living | 1 |
| Address | 2 |
| Age or d.o.b. | 24 |
| Alcohol | 1 |
| Allergies | 15 |
| Angina | 1 |
| Anti-coagulation | 1 |
| Appetite | 2 |
| Attending physician | 9 |
| Bilirubin | 6 |
| Bleeding | 1 |
| Blood chemistry | 3 |
| Blood pressure | 16 |
| Breathlessness | 1 |
| Carbon dioxide level | 2 |
| Cardiac and pulmonary screen | 6 |
| Central venous catheter days | 1 |
| Chest pain | 1 |
| Chronic disease | 1 |
| Comorbidities | 1 |
| Consultation | 2 |
| COPD | 1 |
| Coronary artery disease | 1 |
| Creatinine | 7 |
| Date - Admission | 10 |
| Date - Admission / discharge | 6 |
| Date - Discharge | 3 |
| Depression and anxiety | 4 |
| Diabetes | 1 |
| Diagnosis | 13 |
| Distress | 3 |
| Drowsy | 1 |
| ECG | 3 |
| Electrolyte | 1 |
| Email | 1 |
| Encounter comments | 3 |
| Endoscopies | 1 |
| End stage renal disease | 1 |
| Enjoyment | 1 |
| Equipment | 1 |
| Exam status including specimens | 4 |
| Falls | 1 |
| Falls risk | 1 |
| Family history | 1 |
| Fatigue | 2 |
| Fluid status | 3 |
| Gender / sex | 16 |
| General information | 28 |
| Glasgow Coma Scale | 3 |
| Heart rate | 13 |
| Height | 12 |
| Hospital location | 7 |
| Hospital ward section | 4 |
| Hydration status | 2 |
| Infection status | 6 |
| Influenza status | 3 |
| Information about number of occupied beds | 2 |
| Information about restraints | 1 |
| Insertion date of device | 2 |
| IV lines tubes and drains | 2 |
| Laboratory data | 23 |
| Laboratory data presented in table | 11 |
| LDL | 2 |
| Length of stay | 6 |
| Length of stay - expected | 1 |
| Mean arterial blood pressure | 3 |
| Medical alerts | 13 |
| Medical records | 14 |
| Medication - current | 13 |
| Medication - dose amount | 3 |
| Medication - dose interval | 1 |
| Medication - history | 11 |
| Medication - infusion | 1 |
| Medication - list (from home) | 11 |
| Medication - side effects | 1 |
| Memory | 1 |
| Microbiology | 3 |
| Mood | 2 |
| Mood - Feelings of sadness | 1 |
| Mortality risk | 1 |
| Name | 35 |
| Nausea | 2 |
| Numbness | 1 |
| Oedema | 1 |
| Operation - history | 11 |
| Operation - type | 7 |
| Pacemaker | 1 |
| Pain | 9 |
| Papanicolaou test results | 1 |
| Past medical history | 12 |
| Peripheral oxygen saturations | 5 |
| Peripheral oxygen saturations/Fraction inspired oxygen ratio | 1 |
| Phospate | 1 |
| Physical activity | 5 |
| Physical exam findings | 7 |
| Platelets | 4 |
| Pnuemocystis status | 3 |
| Procedure | 9 |
| Psychological History | 1 |
| Pupillometric Data | 2 |
| Radiation therapy | 1 |
| Radiology results | 7 |
| Readmission status | 3 |
| Referrals | 1 |
| Relationships | 1 |
| Respiratory - history | 13 |
| Respiratory - therapy | 3 |
| Score - Barthel (index) / functional status | 1 |
| Score - Braden | 1 |
| Score - cardiovascular | 1 |
| Score - cirrhosis | 1 |
| Score - Delirium | 1 |
| Section of emergency department | 4 |
| Severity of illness | 3 |
| Sleep | 2 |
| Smoking | 8 |
| Social history | 4 |
| Specific identifying number | 20 |
| Surgery timing | 1 |
| Surgical history | 3 |
| Swelling | 1 |
| Temperature | 3 |
| Total of medication doses | 2 |
| Treatments | 10 |
| Triage | 1 |
| Triglycerides | 1 |
| Type of ICU | 1 |
| Type of urine catheter | 1 |
| Urinalysis | 5 |
| Urinary catheter | 2 |
| Urine catheter days | 1 |
| Vaccines | 3 |
| Ventilator mode | 2 |
| Vital signs | 19 |
| Vomiting | 1 |
| Walking | 1 |
| Weight | 17 |
| Weight gain | 2 |
| Weight loss | 1 |
| Work status | 1 |
