# Supplementary material for: Systematic review of applied usability metrics within usability evaluation methods for hospital electronic healthcare record systems: Metrics and Evaluation Methods for eHealth Systems
Source: J Eval Clin Pract. 2021 May 13;27(6):1403–16. doi: 10.1111/jep.13582 (PMC9438452; doi:10.1111/jep.13582)
Supplement: Supplementary file 6 — Appendix Table S5 Heuristic evaluation [file JEP-27-1403-s010.docx]

**Appendix Table 5.** Heuristic evaluation

| Ref. | **Heuristic checklist** | | | **Importance or severity** | | **Solution** | |
| --- | --- | --- | --- | --- | --- | --- | --- |
|  | Zhang et al.(2003) | Nielsen (1995) | Devine et al. (2014) | Nielsen (1994) | the mean of ratings calculated | Additional feedback how to improve the system | Proposed changes applied |
| [38] | 1 | 0 | 0 | 1 | 1 | 1 | 1 |
| [42] | 0 | 1 | 0 | 1 | 1 | 1 | 0 |
| [45] | 1 | 0 | 1 | 0 | 0 | 1 | 0 |
| [57] | 1 | 0 | 0 | 1 | 0 | 1 | 1 |
| [64] | 0 | 1 | 0 | 1 | 1 | 1 | 1 |
| [68] | 0 | 1 | 0 | 1 | 1 | 1 | 0 |
| [79] | 1 | 0 | 0 | 1 | 1 | 1 | 1 |
| Total | 4 | 3 | 1 | 6 | 5 | 7 | 4 |
