# Supplementary material for: Systematic review of applied usability metrics within usability evaluation methods for hospital electronic healthcare record systems: Metrics and Evaluation Methods for eHealth Systems
Source: J Eval Clin Pract. 2021 May 13;27(6):1403–16. doi: 10.1111/jep.13582 (PMC9438452; doi:10.1111/jep.13582)
Supplement: Supplementary file 7 — Appendix Table S6 Interviews [file JEP-27-1403-s011.docx]

**Appendix Table 6.** Interviews

| **Type of interview** | **When applied** | **Ref** | **Reason of interview’s use** |
| --- | --- | --- | --- |
|  |  |  | **and detailed information** |
|  |  |  | **provided by authors of included studies** |
| Unstructured interview | Before user trial | [47] | -   to collect the type of information needed by ICU physicians |
|  |  |  | -   to understand the use of antibiotics in ICU |
| Follow-up interview | Before user trial | [47] | - to recognise the organisation of the environment and IT infrastructure, |
| Semi-structured interview | Before user trial | [47], [50] | -   to specify users’ needs and to understand the ICU decision-making process |
|  |  |  | Authors divided interview into following sections: |
|  |  |  | - work assignments, |
|  |  |  | - information systems and information/communication flows, |
|  |  |  | - therapy process |
|  |  |  | - decision support for antibiotic use |
|  |  |  | - to enhance the initial clinical prototype and determine major changes in clinical content and data presentation [50] |
| Contextual interview | During user trial | [71] | - watching users while they work to collect information about potential utility of system |
| In-depth debriefing (semi-structured interview) | After user trial | [42] | - getting information about users’ experience using the system including: strengths, weaknesses, opportunities for improvements, and threats of using the software, |
|  |  |  | - interviews based on Normalisation Process Theory to find out how users’ individually and collectively work, |
|  |  |  | - to identify problems which may occur during task performance |
| Pre-structured | After user trial | [59] | - to evaluate technical feasibility from the users' perspective |
| Post-test interview | After user trial | [47] | - debriefing session to gather information about missing data, user expectations for the system and problems that occurred during testing. |
| Semi-structured interview | After user trial | [48], [50], [60], [62], [63], [72] | - understanding user experiences testing the system |
|  |  |  | - perception of system concept and the prototype |
|  |  |  | - user attitudes towards dashboard |
|  |  |  | - understanding physicians’ views regarding clinical information that is hardest to find in the HER, the information most needed for the care of critically ill patients, the perceived utility of key screens and screen features, and the perceived relationship between EHR use and burnout [60] |
|  |  |  | - how clinically challenging the applied scenarios were in a group interview [72] |
|  |  |  | - physician suggestions about requirements, recommendations and areas for tool redesign [50] |
