# Supplementary material for: Systematic review of applied usability metrics within usability evaluation methods for hospital electronic healthcare record systems: Metrics and Evaluation Methods for eHealth Systems
Source: J Eval Clin Pract. 2021 May 13;27(6):1403–16. doi: 10.1111/jep.13582 (PMC9438452; doi:10.1111/jep.13582)
Supplement: Supplementary file 8 — Appendix Table S7 SUS = System Usability Scale, PSSUQ = Post‐Study System Usability Questionnaire, QUIS = User Interaction Satisfaction Questionnaire, CSUQ = Computer Usability Satisfaction Questionnaire, SEQ = a Single Ease Question, OAIQ = Object‐Action Interface Questionnaire, QQ = Qualitative Questionnaire, USQ = User Satisfaction Questionnaire, SUSQ = Subjective User Satisfaction Questionnaire, TAM = TAM, PTSQ = Post‐Task Satisfaction Questionnaire, PTQ = Post‐Test Questionnaire, UQ = Usability Questionnaire, UEQ = Usability Evaluation Questionnaire, PQ = Physician's Questionnaire, TPBT = Three paper‐based tests, 10 item SQ = 10‐item Satisfaction Questionnaire, NASA = NASA Task Load Index, PUS ‐ Perceived Usability Scale, CQ = Clinical Questionnaire, Lee et al Quest = Questionnaire without name in Lee et al. 2017, 2sets of quest = Two sets of questionnaires in Zheng et al. 2013, InterRAI MDS‐HC 2.0 = InterRAI MDS‐HC 2.0, EHRUS = the Electronic Health Record Usability Scale, SAQ = self‐administered questionnaire, PVAS = post‐validation assessment survey, 5pS ‐usability score ‐ 5‐point scale, CSS = The Crew Status Survey [file JEP-27-1403-s005.docx]

| **Ref** | SUS | PSS UQ | QU IS | CSUQ | SEQ | OAIQ | QQ | USQ | SUSQ | TAM | PT SQ | PTQ | UQ | UEQ | PQ | TPBT | 10-item SQ | NASA | PUS | CQ | Lee et al quest | 2 sets of quest | InterRAI MDS-HC 2.0 | EHRUS | S A Q | PVAS | 5 p S | C S S | Number of S/Q | 1 if S/Q applied |
| --- | --- | --- | --- | --- | --- | --- | --- | --- | --- | --- | --- | --- | --- | --- | --- | --- | --- | --- | --- | --- | --- | --- | --- | --- | --- | --- | --- | --- | --- | --- |
| [35] |  |  |  | **1** |  |  |  |  |  |  |  |  |  |  |  |  |  |  |  |  |  |  |  |  |  |  |  |  | 1 | 1 |
| [36] |  | **1** |  |  |  |  |  |  |  |  |  |  |  |  |  |  |  |  |  |  |  |  |  |  |  |  |  |  | 1 | 1 |
| [37] |  |  |  |  |  |  |  |  |  |  |  |  |  |  |  |  |  | **1** |  |  |  |  |  |  |  |  |  |  | 1 | 1 |
| [38] |  |  |  |  |  |  |  |  |  |  |  |  |  |  |  |  |  |  |  |  |  |  |  |  |  |  |  |  | 0 | 0 |
| [39] | **1** |  |  |  |  |  |  |  |  |  |  |  |  |  |  |  |  | **1** |  |  |  |  |  |  |  |  |  |  | 2 | 1 |
| [40] |  |  |  |  |  |  |  |  |  |  |  |  | **1** |  |  |  |  |  |  |  |  |  |  |  |  |  |  |  | 1 | 1 |
| [41] |  |  |  |  |  |  |  |  |  |  |  |  |  |  |  |  |  |  |  |  |  |  |  |  |  |  |  |  | 0 | 0 |
| [42] |  |  |  |  |  |  |  |  |  | **1** |  |  |  |  |  |  |  |  |  |  |  |  | **1** |  |  |  |  |  | 2 | 1 |
| [43] | **1** |  |  |  |  | **1** |  |  |  |  |  |  |  |  |  |  |  |  |  |  |  |  |  |  |  |  |  |  | 2 | 1 |
| [44] | **1** |  |  |  |  |  |  | **1** |  |  |  |  |  |  |  |  |  | **1** |  | **1** |  |  |  |  |  |  |  |  | 4 | 1 |
| [45] |  | **1** |  |  |  |  |  |  |  |  |  |  |  |  |  |  |  |  |  |  |  |  |  |  |  |  |  |  | 1 | 1 |
| [46] |  |  |  |  |  |  |  |  |  |  |  |  |  |  |  |  |  |  | **1** |  |  |  |  |  |  |  |  |  | 1 | 1 |
| [47] | **1** |  |  |  |  |  |  |  |  |  |  |  |  |  |  |  |  |  |  |  |  |  |  |  |  |  |  |  | 1 | 1 |
| [48] |  |  |  |  |  |  |  |  |  |  |  |  |  | **1** |  |  |  |  |  |  |  |  |  |  |  |  |  |  | 1 | 1 |
| [49] | **1** |  |  |  |  |  |  |  |  |  |  |  |  |  |  |  |  |  |  |  |  |  |  |  |  |  |  |  | 1 | 1 |
| [50] | **1** |  |  |  |  |  |  |  |  |  |  |  |  |  |  |  |  |  |  |  |  |  |  | **1** |  |  |  |  | 2 | 1 |
| [51] | **1** |  |  |  |  |  |  |  |  |  |  |  |  |  |  |  |  |  |  |  |  |  |  |  |  |  |  |  | 1 | 1 |
| [52] | **1** |  |  |  |  |  |  |  |  |  |  |  |  |  |  |  |  |  |  |  |  |  |  |  |  |  |  |  | 1 | 1 |
| [53] |  |  |  |  |  |  |  |  |  |  | **1** |  |  |  |  |  |  |  |  |  |  |  |  |  |  |  |  |  | 1 | 1 |
| [54] |  |  |  |  |  |  |  |  |  |  |  |  |  |  | **1** |  |  |  |  |  |  |  |  |  |  |  |  |  | 1 | 1 |
| [55] |  |  | **1** |  |  |  |  |  |  |  |  |  |  |  |  |  |  |  |  |  |  |  |  |  |  |  |  |  | 1 | 1 |
| [56] | **1** |  |  |  | **1** |  |  |  |  |  |  |  |  |  |  |  |  |  |  |  |  |  |  |  |  |  |  |  | 2 | 1 |
| [57] |  |  |  |  |  |  |  |  |  |  |  |  |  |  |  |  |  |  |  |  |  |  |  |  |  |  |  |  | 0 | 0 |
| [58] |  |  |  |  |  |  |  |  |  |  |  |  |  |  |  |  |  |  |  |  |  |  |  |  |  |  |  |  | 0 | 0 |
| [59] |  |  | **1** | **1** |  |  |  |  |  |  |  |  |  |  |  |  |  |  |  |  |  |  |  |  |  |  |  |  | 2 | 1 |
| [60] | **1** |  |  |  |  |  |  |  |  |  |  |  |  |  |  |  |  |  |  |  |  |  |  |  | **1** |  |  |  | 2 | 1 |
| [61] | **1** |  | **1** |  |  |  |  |  |  |  |  |  |  |  |  |  |  | **1** |  |  |  |  |  |  |  |  |  |  | 3 | 1 |
| [62] |  |  |  | **1** |  |  |  |  |  |  |  |  |  |  |  |  |  |  |  |  |  |  |  |  |  |  |  |  | 1 | 1 |
| [63] | **1** |  |  |  |  |  |  |  |  |  |  |  |  |  |  |  |  |  |  |  |  |  |  |  |  |  |  |  | 1 | 1 |
| [64] |  |  |  |  |  |  |  |  |  |  |  |  |  |  |  |  |  |  |  |  |  |  |  |  |  |  |  |  | 0 | 0 |
| [65] |  |  |  |  |  |  |  |  |  |  |  |  |  |  |  |  |  |  |  |  |  |  |  |  |  |  |  |  | 0 | 0 |
| [66] |  |  |  |  |  |  |  |  |  |  |  |  |  |  |  |  |  |  |  |  | **1** |  |  |  |  |  |  |  | 1 | 1 |
| [67] |  |  |  |  |  |  |  |  |  |  |  | **1** |  |  |  |  |  |  |  |  |  |  |  |  |  |  |  |  | 1 | 1 |
| [68] |  |  |  |  |  |  |  |  |  |  |  |  |  |  |  |  |  | **1** |  |  |  |  |  |  |  |  |  | **1** | 2 | 1 |
| [69] |  |  |  |  |  |  |  |  |  |  |  |  |  |  |  |  |  |  |  |  |  |  |  |  |  |  |  |  | 0 | 0 |
| [70] | **1** |  |  |  |  |  |  |  |  |  |  |  |  |  |  |  |  |  |  |  |  |  |  |  |  |  |  |  | 1 | 1 |
| [71] | **1** |  |  |  |  |  |  |  |  |  |  |  |  |  |  |  |  |  |  |  |  |  |  |  |  |  |  |  | 1 | 1 |
| [72] |  | **1** |  |  |  |  |  |  |  |  |  |  |  |  |  |  | **1** |  |  |  |  |  |  |  |  |  |  |  | 2 | 1 |
| [73] |  |  |  |  |  |  |  |  |  |  |  |  |  |  |  |  |  |  |  |  |  |  |  |  |  | **1** |  |  | 1 | 1 |
| [74] |  |  |  |  |  |  |  |  | **1** |  |  |  |  |  |  |  |  |  |  |  |  |  |  |  |  |  |  |  | 1 | 1 |
| [75] | **1** |  |  |  |  |  | **1** |  |  |  |  |  |  |  |  |  |  |  |  |  |  |  |  |  |  |  |  |  | 2 | 1 |
| [76] | **1** | **1** |  |  |  |  |  |  |  |  |  |  |  |  |  |  |  |  |  |  |  |  |  |  |  |  |  |  | 2 | 1 |
| [77] |  |  |  |  |  |  |  |  |  |  |  |  |  |  |  |  |  |  |  |  |  |  |  |  |  |  | **1** |  | 1 | 1 |
| [78] |  |  | **1** |  |  |  |  |  |  |  |  |  |  |  |  |  |  |  |  |  |  |  |  |  |  |  |  |  | 1 | 1 |
| [79] |  | **1** |  |  |  |  |  |  |  |  |  |  |  |  |  |  |  | **1** |  |  |  |  |  |  |  |  |  |  | 2 | 1 |
| [80] |  |  |  |  |  |  |  |  |  |  |  |  |  |  |  |  |  |  |  |  |  |  |  |  |  |  |  |  | 0 | 0 |
| [81] |  |  |  |  |  |  |  |  |  |  |  |  |  |  |  | **1** |  |  |  |  |  |  |  |  |  |  |  |  | 1 | 1 |
| [82] |  |  |  |  |  |  |  |  |  |  |  |  |  |  |  |  |  |  |  |  |  |  |  |  |  |  |  |  | 0 | 0 |
| [83] |  |  |  |  |  |  |  |  |  |  |  |  |  |  |  |  |  |  |  |  |  |  |  |  |  |  |  |  | 0 | 0 |
| [84] |  |  |  |  |  |  |  |  |  |  |  |  |  |  |  |  |  |  |  |  |  | **1** |  |  |  |  |  |  | 1 | 1 |
| [85] |  |  |  |  |  |  |  |  |  |  |  |  |  |  |  |  |  |  |  |  |  |  |  |  |  |  |  |  | 0 | 0 |
|  | 16 | 5 | 4 | 3 | 1 | 1 | 1 | 1 | 1 | 1 | 1 | 1 | 1 | 1 | 1 | 1 | 1 | 6 | 1 | 1 | 1 | 1 | 1 | 1 | 1 | 1 | 1 | 1 | 57 | 40 |
|  | SUS | PSS UQ | QU IS | CSUQ | SEQ | OAIQ | QQ | USQ | SUSQ | TAM | PT SQ | PTQ | UQ | UEQ | PQ | TPBT | 10-item SQ | NASA | PUS | CQ | Lee et al Quest | 2 sets of quest | InterRAI MDS-HC 2.0 | EHRUS | SAQ | PVAS | 5 p S | CS S |  |  |

**SUS** = System Usability Scale, **PSSUQ** = Post-Study System Usability Questionnaire, **QUIS** = User Interaction Satisfaction Questionnaire, **CSUQ** = Computer Usability Satisfaction Questionnaire, **SEQ** = a Single Ease Question, **OAIQ** = Object-Action Interface Questionnaire, **QQ** = Qualitative Questionnaire, **USQ** = User Satisfaction Questionnaire, **SUSQ** = Subjective User Satisfaction Questionnaire, **TAM** =TAM, **PTSQ** = Post-Task Satisfaction Questionnaire, **PTQ**=Post-Test Questionnaire, **UQ** = Usability Questionnaire, **UEQ** = Usability Evaluation Questionnaire, **PQ** = Physician’s Questionnaire, **TPBT** = Three paper-based tests, **10 item SQ** = 10-item Satisfaction Questionnaire, **NASA** = NASA Task Load Index, **PUS** – Perceived Usability Scale, **CQ** = Clinical Questionnaire, **Lee et al Quest** = Questionnaire without name in Lee et al. 2017, **2sets of quest** = Two sets of questionnaires in Zheng et al. 2013, **InterRAI MDS-HC 2.0** = InterRAI MDS-HC 2.0, **EHRUS** = the Electronic Health Record Usability Scale, **SAQ** = self-administered questionnaire, **PVAS** = post-validation assessment survey, **5pS** -usability score - 5-point scale, **CSS**= The Crew Status Survey
