# Supplementary material for: Systematic review of applied usability metrics within usability evaluation methods for hospital electronic healthcare record systems: Metrics and Evaluation Methods for eHealth Systems
Source: J Eval Clin Pract. 2021 May 13;27(6):1403–16. doi: 10.1111/jep.13582 (PMC9438452; doi:10.1111/jep.13582)
Supplement: Supplementary file 9 — Appendix Table S8 How usability metrics results were reported ‐ with given number of studies, which used the selected measurement techniques S/Q ‐ Survey/Questionnaire, UT ‐ User Trial, CW‐HE ‐ Cognitive Walkthrough, I ‐ Interview, [file JEP-27-1403-s001.docx]

**Appendix Table 8**. How usability metrics results were reported – with given number of studies, which used the selected measurement techniques

| **METRIC** | **METRICS MEASUREMENT TECHNIQUES** | **Number of cases** |
| --- | --- | --- |
| **USEFULNESS**  reported as: | System usefulness average score (questions 1 to 6) of PSSUQ - (S/Q) | 5 |
|  | User comments related to the system usefulness in the clinical practice - (UT)/(I) | 5 |
|  | User comments related to the usefulness of layout - (UT) | 1 |
|  | User comments related to the usefulness as technology quality improvement - (UT) | 1 |
|  | Positive Snippets' related to 'Usefulness of the decision support tool' area of evaluation - (I) | 1 |
|  | User opinion about usefulness of key screens and screen features - (I) | 1 |
|  | Perceived usefulness as TAM questionnaire results - (S/Q) | 1 |
|  | Qualitative questionnaire results [75] - (S/Q) | 1 |
|  | User satisfaction questionnaire results [44] - (S/Q) | 1 |
|  | Total mean scores for 7 work system-usefulness-related dimensions [65] - (S/Q) | 1 |
|  | 5-point scale results for assessment of usefulness of specific tasks completion [76] - (S/Q) | 1 |
|  | Result of the Electronic Record Health Usability Scale - usefulness of the tool for decision-making [50] - (S/Q) | 1 |
| **SATISFACTION** reported as: | Questionnaire result | 31 |
|  | Identified general comments related to the system satisfaction - (UT) | 10 |
|  | Number of positive comments - (UT) | 4 |
|  | Number of negative comments - (UT) | 4 |
|  | User preference: number of participants who indicated redesigned/original display as easier to work with or indicated that they were both the same - (UT) | 1 |
|  | Perceived barriers and facilitators (quotes) - (UT)/(I) | 2 |
|  | User comments from NPT-driven SWOT analysis interviews [42] (Brown et al 2018) - (I) | 1 |
|  | Number of positive comments related to heuristic themes [41] - (CW-HE) | 1 |
| **EFFICIENCY** reported as: | (New time) - Measured time to complete task using new or improved system - (UT) | 27 |
|  | (Old time) - Measured time to complete task using old or previous system - (UT) | 13 |
|  | Percentage of tasks completed without error - (UT) | 2 |
|  | Number of clicks required to complete tasks - (UT) | 11 |
|  | Clicks compared to optimal paths - (UT) | 5 |
|  | Time of patient state assessment - (UT) | 2 |
|  | Perceived speed of entry (Faster than expected, just as easy as expected, harder than expected) [40] - (S/Q) | 1 |
|  | Less handwriting [43] - (S/Q) | 1 |
|  | More time for patients [43] - (S/Q) | 1 |
| **EFFECTIVENESS** reported as: | Number of successfully completed tasks (UT) | 8 |
|  | Percentage of correct responses - (UT) | 4 |
|  | Percentage of participants able to complete tasks - (UT) | 3 |
|  | Percentage of task completed with no difficulty - (UT) | 1 |
|  | Information about task completion (succeeded vs failed) for every user - (UT) | 1 |
|  | Number of incomplete tasks - (UT) | 1 |
|  | Number of incomplete data - (UT) | 1 |
|  | Result of responses of users who were able to completely or mostly enter the assessment data [40] - (S/Q) | 1 |
|  | Perceived ease of data entry (easier than expected, just as easy as expected, harder than expected) [40] - (S/Q) | 1 |
|  | Perceived by users whether the screen design facilitated your data entry Yes/No [40] - (S/Q) | 1 |
|  | Participants accuracy responses comparison (M, SD, Mdn, IQR reported) - (UT) | 1 |
|  | Users' navigation pathways for old and new system - (UT) | 1 |
|  | Number of instances of recording incorrect data - (UT) | 1 |
|  | Result of survey H section responses - expectation for work effectiveness [65] - (S/Q) | 1 |
|  | Percentage of errors (UT) | 7 |
|  | Errors in details are described in Errors section |  |
| **ERRORS** reported as: | Number of Errors (UT) | 4 |
|  | Percentage of Errors (UT) | 6 |
|  | Percentage of tasks completed without errors (UT) | 1 |
|  | Types of errors - (UT) | 5 |
|  | Reason for errors - (UT) | 1 |
|  | How easily users can recover from the errors - (UT) | 1 |
|  | No errors occur during system use - 5-point scale used - (S/Q) | 1 |
|  | Number of missed events for every completed task [78] - (UT) | 1 |
|  | Number of false events for every completed task [78] - (UT) | 1 |
|  | Number and % of failure opportunities - (UT) | 1 |
|  | Visual attention heatmaps and fixation sequences - (UT) | 2 |
|  | Errors rate with completion success rate time-on-task [70] - (UT) | 1 |
|  | The overall use error rating [66] - (UT) | 1 |
|  | Interview's findings which provided insights into errors observed during user observation (I) | 1 |
|  | User error rating: users task completion with no hint/clarification or reminders [66] - (UT) | 1 |
|  | User error rating: users task completion with no hint/clarification or reminders [66] - (UT) | 1 |
|  | User error rating: users task completion with one hint [66] - (UT) | 1 |
|  | User error rating: number of users who did not completed task despite several hints [66] - (UT) | 1 |
|  | Average of completed tasks for all participants [66] - (UT) | 1 |
|  | Number of missed data [63] - (UT) | 1 |
|  | Number of instances recorded incorrectly [63] - (UT) | 1 |
|  | Number of wrong buttons use, observed errors during system test [55] - (UT) | 1 |
|  | Heuristics results using heuristic checklist of Zhang et al. [94] | 1 |
| **MEMORABILITY** reported as: | Result of questionnaire on how easy to learn the new system was - (S/Q) | 10 |
| **LEARNABILITY** reported as: | Comparison time of current with previous task - (UT) | 4 |

**S/Q** – Survey/Questionnaire, **UT** – User Trial, **CW-HE** – Cognitive Walkthrough, **I** – Interview,
