# Supplementary material for: Systematic review of applied usability metrics within usability evaluation methods for hospital electronic healthcare record systems: Metrics and Evaluation Methods for eHealth Systems
Source: J Eval Clin Pract. 2021 May 13;27(6):1403–16. doi: 10.1111/jep.13582 (PMC9438452; doi:10.1111/jep.13582)
Supplement: Supplementary file 10 — Appendix Table S9 Quality Assessment results (in %) using the Downs & Black checklists [file JEP-27-1403-s008.docx]

**Appendix Table 9.** Quality Assessment results (in %) using the Downs & Black checklists

| Reference | Downs & Black checklist | Modified Downs & Black checklist |
| --- | --- | --- |
| 35 | 31 | 50 |
| 36 | 34 | 80 |
| 37 | 34 | 90 |
| 38 | 25 | 70 |
| 39 | 34 | 90 |
| 40 | 28 | 60 |
| 41 | 25 | 80 |
| 42 | 38 | 80 |
| 43 | 38 | 80 |
| 44 | 28 | 80 |
| 45 | 38 | 70 |
| 46 | 34 | 70 |
| 47 | 28 | 70 |
| 48 | 31 | 60 |
| 49 | 28 | 70 |
| 50 | 28 | 90 |
| 51 | 34 | 80 |
| 52 | 41 | 80 |
| 53 | 34 | 70 |
| 54 | 22 | 30 |
| 55 | 31 | 70 |
| 56 | 28 | 70 |
| 57 | 22 | 40 |
| 58 | 31 | 70 |
| 59 | 38 | 70 |
| 60 | 28 | 70 |
| 61 | 41 | 80 |
| 62 | 31 | 40 |
| 63 | 34 | 70 |
| 64 | 28 | 70 |
| 65 | 41 | 90 |
| 66 | 31 | 70 |
| 67 | 38 | 60 |
| 68 | 25 | 70 |
| 69 | 31 | 80 |
| 70 | 25 | 60 |
| 71 | 13 | 10 |
| 72 | 38 | 90 |
| 73 | 28 | 40 |
| 74 | 31 | 70 |
| 75 | 22 | 60 |
| 76 | 28 | 80 |
| 77 | 34 | 70 |
| 78 | 38 | 90 |
| 79 | 41 | 70 |
| 80 | 19 | 40 |
| 81 | 25 | 70 |
| 82 | 22 | 60 |
| 83 | 25 | 50 |
| 84 | 38 | 80 |
| 85 | 38 | 60 |
