# Supplementary material for: Systematic review of applied usability metrics within usability evaluation methods for hospital electronic healthcare record systems: Metrics and Evaluation Methods for eHealth Systems
Source: J Eval Clin Pract. 2021 May 13;27(6):1403–16. doi: 10.1111/jep.13582 (PMC9438452; doi:10.1111/jep.13582)
Supplement: Supplementary file 11 — Appendix Table S10 Domains within the Downs & Black Checklist The % score of each included study for each domain. [file JEP-27-1403-s002.docx]

**Appendix Table 10**. Domains within the Downs & Black Checklist

| Reporting | 54% |
| --- | --- |
| External validity | 7% |
| Internal validity - bias | 43% |
| Internal validity – confounding | 21% |
| Power | 0% |

The % score of each included study for each domain.
